# Replicating CD Nanogrooves onto PDMS to Guide Nanowire Growth for Monolithic Flexible Photodetectors with High Bending‐Stable UV–vis–NIR Photoresponse

**DOI:** 10.1002/advs.202403870

**Published:** 2024-06-20

**Authors:** Hanyu Liu, Wei Zhou, Xiangtao Chen, Pingyang Huang, Xingyu Wang, Guofu Zhou, Jinyou Xu

**Affiliations:** ^1^ Guangdong Provincial Key Laboratory of Optical Information Materials and Technology & Institute of Electronic Paper Displays South China Academy of Advanced Optoelectronics South China Normal University Guangzhou 510006 P. R. China

**Keywords:** array, flexible photodetectors, monolithic integration, nanowires, organic semiconductors

## Abstract

Guided nanowires grown on polymer surfaces facilitate their seamless integration as flexible devices without post‐growth processing steps. However, this is challenging due to the inability of polymer films to provide the required lattice‐matching effect. In this work, this challenge is addressed by replicating highly aligned nanogrooves from a compact disc (CD) onto a casted flexible polydimethylsiloxane (PDMS) surface. Leveraging the replicated nanogrooves, copper hexadecafluorophthalocyanine (F_16_CuPc) and various metal phthalocyanines are guided into large‐area, self‐aligned nanowires. Subsequently, by employing specifically designed shadow masks during electrode deposition, these nanowires are seamlessly integrated as either a monolithic flexible photodetector with a large sensing area or on‐chip flexible photodetector arrays. The resulting flexible photodetectors exhibit millisecond and long‐term stable response to UV–vis–NIR light. Notably, they demonstrate exceptional bending stability, retaining stable and sensitive photoresponse even at a curvature radius as low as 0.5 cm and after enduring 1000 bending cycles. Furthermore, the photodetector array showcases consistent sensitivity and response speed across the entire array. This work not only proves the viability of guided nanowire growth on flexible polymer surfaces by replicating CD nanogrooves but also underscores the potential for large‐scale monolithic integration of guided nanowires as flexible devices.

## Graphical abstract

A general method is proposed for the guided growth of nanowires on flexible surfaces by replicating nanogrooves from commercial CDs onto casted PDMS films, facilitating seamless integration of nanowires as flexible photodetectors on the growth film. The photodetectors exhibit UV–vis–NIR photoresponse and high bending stability. This in situ integration strategy represents significant advances in implementing flexible electronics from nanowire arrays.

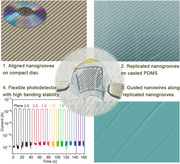

## 1 Introduction

Nanowires, characterized by their distinctive geometry and exceptional flexibility, hold significant promise as building blocks for the implementation of flexible functional devices capable of enduring bending, twisting, and stretching without compromising their functionality.^[^ ^1^, ^2^, ^3^, ^4^, ^5^, ^6^, ^7^, ^8^, ^9^, ^10^, ^11^ ^]^ The assembly of large numbers of nanowires into flexible devices presents new opportunities for the development of wearable/foldable electronics,^[^ ^2^, ^3^, ^8^, ^12^ ^]^ flexible sensors and imaging,^[^ ^1^, ^4^, ^10^, ^13^, ^14^, ^15^ ^]^ and the emerging stretchable artificial synapses,^[^ ^16^, ^17^ ^]^ while also enabling device miniaturization for lightweight and compact designs.^[^ ^11^, ^18^ ^]^ However, the intricate process of transferring nanowires onto flexible target films frequently leads to annoying damage and contamination, potentially degrading performance. Consequently, developing methods to grow large arrays of ordered nanowires directly on flexible surfaces is crucial for enhancing both efficiency and technological innovation.^[^ ^1^, ^2^, ^6^, ^14^ ^]^

Various principles and mechanisms have been proposed to facilitate the self‐aligned growth of nanowires, leveraging concepts such as out‐of‐plane epitaxial growth,^[^ ^9^, ^10^, ^19^ ^]^ in‐plane epitaxial growth,^[^ ^20^, ^21^, ^22^, ^23^, ^24^ ^]^ and graphoepitaxial growth.^[^ ^7^, ^8^, ^25^, ^26^, ^27^ ^]^ To date, a variety of highly aligned inorganic nanowires (i.e., GaN,^[^ ^28^ ^]^ II‐VI,^[^ ^29^, ^30^, ^31^, ^32^ ^]^ CsPbX_3_,^[^ ^33^, ^34^, ^35^ ^]^ tellurium^[^ ^36^ ^]^) and organic nanowires (e.g., metal phthalocyanines, Alq3)^[^ ^37^, ^38^, ^39^, ^40^ ^]^ have been guided on various rigid single‐crystal surfaces including sapphire,^[^ ^29^, ^33^, ^36^ ^]^ GaN,^[^ ^41^ ^]^ SiC,^[^ ^28^ ^]^ spinel,^[^ ^42^ ^]^ silicon,^[^ ^43^ ^]^ LaAlO_3_,^[^ ^32^ ^]^ mica.^[^ ^34^ ^]^ These methods generally depend on a minimal lattice mismatch between the growing nanowires and the underlying substrates, which poses a challenge when endeavoring to grow self‐aligned nanowires on the amorphous surfaces such as flexible polymer films. Despite efforts to grow III–V nanowires (e.g., InAs and InP,^[^ ^44^ ^]^ GaAs,^[^ ^45^ ^]^ InSb^[^ ^46^ ^]^) directly on flexible polyimide (PI) surfaces, they all stand freely with random orientations. Therefore, there is an urgent need to explore innovative approaches for controlling growth orientation without resorting to lattice‐matching effect.^[^ ^12^, ^15^ ^]^ In a recent breakthrough, we demonstrated the first horizontally oriented growth of nanowires directly on a flexible PI surface. The key to success relies on replicating the periodic V‐shaped nanogrooves on an M‐plane sapphire surface, which formed spontaneously after high‐temperature annealing (1600 °C, 10 h), onto the PI surface through a hot stamping process (220 °C, 100 MPa, 2 h).^[^ ^39^ ^]^ Nevertheless, the high pressure needed for the hot stamping process makes it difficult to apply to other master templates.

In this study, we successfully achieved guided growth of organic molecular nanowires by replicating the highly aligned nanogrooves from a commercially available compact disc (CD) onto a casted flexible polydimethylsiloxane (PDMS) surface at 60 °C. The precise alignment of these nanowires facilitated their seamless integration as monolithic flexible photodetectors. By adjusting the electrode configuration, numerous nanowires were bridged to form either a monolithic photodetector with a substantial sensing area or an on‐chip photodetector array with multiple aligned cells. This in situ fabrication strategy ensures that the resultant photodetectors have a stable photoresponse over a wide UV–vis–NIR spectrum, even under extreme bending conditions and a large number of bending cycles. This study not only advances the field of flexible electronics but also demonstrates a scalable approach to fabricating high‐performance flexible photodetectors.

## 2 Results and Discussion

**Figure 1:**
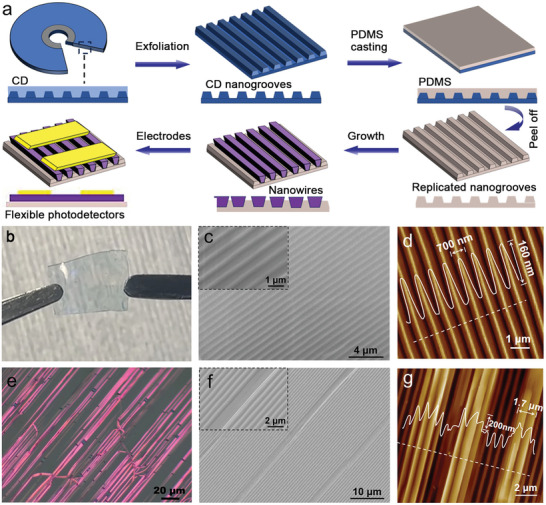
a) Schematic illustration of the key steps. b) Digital photography of the flexible PDMS film grown with nanowires. c) SEM and d) AFM image of the casted PDMS surface with replicated CD nanogrooves. e) Optical microscopy, f) SEM, and g) AFM image of the guided F_16_CuPc nanowires on the PDMS film.

Commercially available CDs contain two polycarbonate plates patterned with a spiral track of grooves and ridges winding from the disc's inner hole to its outer edge (Figure S1, Supporting Information). As shown schematically in **Figure** **1**a, the bottom polycarbonate layer of as‐received CD is first exfoliated to serve as the host template. Next, a PDMS precursor solution is casted on the exfoliated CD polycarbonate masters and subsequently cured at 60 °C in a vacuum drying oven. After mechanically peeling the cured PDMS layer off the CD masters (Figure 1b), the transparent, mechanically and chemically robust PDMS surface inherits the morphology of the periodic CD grooves. As shown in Figure 1c,d, the replicated grooves have a consistent pitch of 700 nm and depth of 160 nm, mirroring the features of the CD grooves (Figure S1, Supporting Information). Compared to the previous hot stamping process with high‐temperature annealed sapphire templates,^[^ ^39^ ^]^ the requirements for the casting process are significantly simpler. In addition, the width and orientation of the nanogrooves on a low‐cost CD surface are more uniform than on a single‐crystal sapphire surface. Furthermore, the CD nanogrooves can also be replicated on other flexible films, such as polyvinyl alcohol (PVA), through this casting process (Figure S2, Supporting Information). This patterned PDMS film is then used as a substrate for the growth of nanowires in a vapor phase atmosphere. The graphoepitaxial effect of the replicated nanogrooves^[^ ^37^, ^38^, ^39^, ^47^, ^48^, ^49^, ^50^ ^]^ guides the growth of nanowires side by side on these films, resulting in the formation of self‐aligned nanowires conducive for subsequent device integration. For example, Figure 1e and Figure S3 (Supporting Information) show that most F_16_CuPc nanowires grown on this hydrophobic PDMS film are well aligned side by side. In contrast, free‐lying F_16_CuPc nanowires lacking preferred orientations are obtained on the hydrophobic and hydrophilic flat PDMS films without the replicated CD nanogrooves (Figures S3,S4, Supporting Information). Scanning electron microscopy (SEM) images confirm that the aligned orientation is dictated by the replicated nanogrooves (Figure 1f). These findings underscore the pivotal role of the replicated hydrophobic nanogrooves in guiding nanowires to grow along a consistent orientation. The high‐density aligned F_16_CuPc nanowires are typically a few microns wide (Figure 1e) and tens of microns long (Figure 1f), forming a nanobelt structure. The thickness of most F_16_CuPc nanowires is 100–200 nm, as determined by the atomic force microscopy (AFM) image (Figure 1g). Noteworthy observations from these morphological characterizations include the tendency for the width of the guided nanobelts to exceed that of the replicated nanogrooves, along with the replication of the underlying nanogroove morphology on the top surfaces of the nanobelts. These observations highlight the pronounced graphoepitaxial influence of the replicated nanogrooves in determining the growth orientation of nanowires.

**Figure 2:**
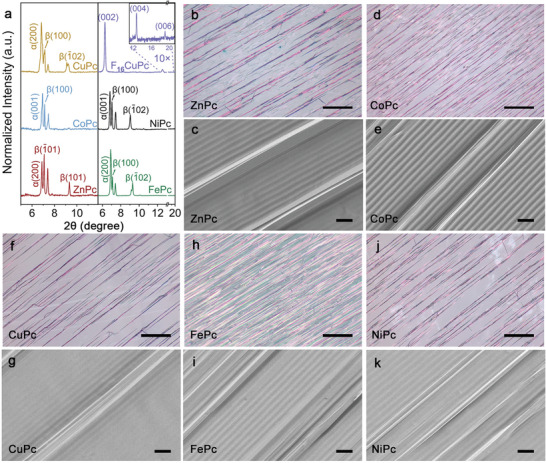
Guided MPc (M = Zn, Co, Cu, Fe, Ni) nanowires by the replicated CD nanogrooves on flexible PDMS films. a) XRD. b,d,f,h,j) Optical microscope images, scale bars are 100 µm. c,e,g,i,k) SEM images, scale bars are 2 µm.

In the X‐ray diffraction (XRD) analysis of the guided F_16_CuPc nanobelts, only three distinct peaks were observed (**Figure** **2**a), a significantly lower number compared to the diffraction peaks of the evaporated powder (Figure S5, Supporting Information). These peaks are indexed into the Bragg reflections of the (002), (004), and (006) crystallographic planes of the orthorhombic F_16_CuPc crystal (CCDC No.698474). They all belong to the {001} lattice group, thereby most F_16_CuPc nanobelts exhibit high single‐crystallinity and the same top surface consisting of (001) planes. This finding is in agreement with previous research on F_16_CuPc nanobelts grown along V‐shaped nanogrooves on annealed M‐plane sapphire.^[^ ^37^ ^]^ Therefore, it makes sense to deduce that most of these nanobelts have the same growth axis of [100]. The consistent crystallographic growth orientation was further verified using a cross polarized optical microscope (POM), a powerful non‐contact tool for quick and large‐scale identification of optically anisotropic materials.^[^ ^39^, ^40^ ^]^ As shown in Figure S6 (Supporting Information), the in situ POM images reveal that most self‐aligned F_16_CuPc nanowires show the same angle‐dependent brightness variation, confirming their high single‐crystallinity. All Raman shift peaks from the nanobelts match well with those of the precursor powder, indicating that these peaks originate from the molecular vibrations of the F_16_CuPc crystal (Figure S7, Supporting Information) and no other impurities were formed during the nanobelt growth. In view of the morphological and structural characterization results, the replicated nanogrooves facilitate the guided growth while maintaining a high crystallinity and a consist growth axis.

Note that there is no lattice matching effect between the guided F_16_CuPc nanobelts and the amorphous PDMS. Consequently, it is anticipated that similar molecules can also be guided into highly aligned nanowires on such patterned PDMS films. The generality of this guided growth was further validated by the successfully guiding nanowires using other p‐type metal phthalocyanine molecules (MPc, M = Zn, Co, Cu, Fe, Ni). As shown in Figure 2b–k, these nanowires are also well aligned along the replicated nanogrooves. In contrast to the guided F_16_CuPc nanobelts, these nanowires are characterized by a smaller width and a lower presence of unwanted disordered structures. To further reduce such disordered nanowires, however, labor‐intensive optimization of growth parameters is necessary. Interestingly, even when the nanowires reach extremely high coverage of the substrate (Figure 2h), they still maintain a nanowire structure rather than merging into a continuous film. Furthermore, the sharp peaks observed in the XRD spectra of MPc nanowires are indexed into the diffractions of different lattice groups (Figure 2a), suggesting that the top surfaces of these nanowires are composed of different lattice planes. As confirmed by the SEM images in Figure 2, different nanowire surfaces were identified, while the same top surface was observed for F_16_CuPc nanobelts. Anyhow, the sharp diffraction peaks indicate that these nanowires also possess a high degree of single crystallinity.

**Figure 3:**
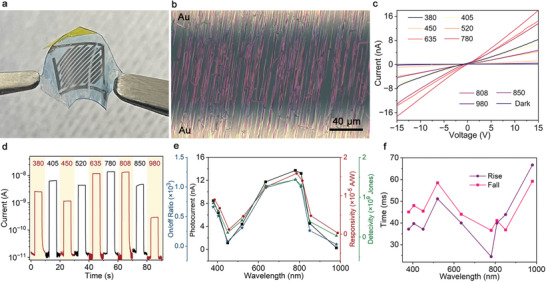
Monolithic flexible photodetectors integrated with large‐area guided F_16_CuPc nanobelts on PDMS films and their wavelength‐dependent photoresponse. a) Digital photography of the photodetector with interdigital electrodes. b) Optical microscope image between electrode gap. c) Voltage‐current curves in response to constant light intensities of 25.5 mW cm^−2^. d) Photocurrent temporal transients at 15 V bias, the light intensity is 25.5 mW cm^−2^ and modulated with 0.1 Hz on/off frequency. e) The extracted net photocurrents, on/off current ratios, responsivities, and detectivities from (d). f) Rise and fall times derived from (d).

The above discussion demonstrates the feasibility to guide organic molecules into highly aligned nanowires along replicated CD nanogrooves on flexible PDMS films. The remarkable alignment of such nanowires facilitates their seamless integration as flexible devices directly on the substrate where they grow. This in situ integration eliminates the need for complicated post‐growth processing steps like dispersion, transfer, and assembly, along with the associated risk of contamination. As depicted in **Figure** **3**a, a flexible photodetector with gold interfinger electrodes was successfully obtained. Numerous highly aligned F_16_CuPc nanowires bridge the gaps between adjacent electrodes (120 µm, Figure 3b), forming a monolithic photodetector characterized by a substantial sensing area. The length and morphology of these nanowires are preserved after the electrode deposition, establishing a robust connection between the electrodes. These observations underscore the potential benefits of this in situ device integration compared to ex situ assembly methods, which typically entail additional transfer and alignment steps after nanowire growth, potentially leading to structural damage and chemical contamination. The preserved morphology and quality of the nanowires offer a promising solution to the challenge of forming reliable electrical contact between most organic semiconductors and deposited metal layers.

When the device is exposed to various monochromatic lights within the UV–vis–NIR range (380, 405, 450, 520, 635, 780, 808, 850, 980 nm), the current flowing through the nanowires shows a linear correlation with the applied voltage (Figure 3c), indicating consistent ohmic contact between the F_16_CuPc nanowires and the deposited gold electrodes. The slope of these curves, representing the overall conductivity of these nanowires, exhibits significant fluctuations with the changes in light wavelength (Figure S8, Supporting Information). The significant variation in conductivity with light wavelength is a crucial characteristic that benefits photodetectors in several ways, such as wavelength selectivity, multiple‐spectral and broadband detection, enhanced sensitivity, improved signal‐to‐noise ratio, which enables the design of more sensitive, selective, and versatile photodetectors for a wide range of applications. The varied photoresponse in the broad UV–vis–NIR band is consistent with the varied but broad absorption of F_16_CuPc nanowires (Figure S9, Supporting Information). The light wavelength‐dependent response of these F_16_CuPc nanowires was further confirmed in the plot of photocurrent temporal transients. As shown in Figure 3d,e, varying photocurrents on the order of 10^−8^ A were recorded when identical incident power density was utilized. In contrast, the dark currents consistently remained as low as 10^−11^ A. Therefore, the on/off current ratio of the detector reaches up to 103, surpassing by at least an order of magnitude the performance of previous photodetectors constructed from organic nanowires.^[^ ^37^, ^38^, ^50^ ^]^

Responsivity (*R*) and detectivity (*D*) are two important parameters used to characterize the performance of a photodetector. The former is a measure of how efficiently a photodetector converts incident light into an electrical signal, and the later is a figure of merit that quantifies the ability of a photodetector to detect weak optical signals in the presence of noise. They are estimated using Equations (1) and (2), respectively.

(1) $$R=\frac{I_{\mathrm{ph}}}{P*A}$$

(2) $$D=\frac{R}{\sqrt{2e\frac{I_{\mathrm{dark}}}{A}}}$$

where *I*_ph_, *P*, *e*, *I*_dark_, and *A* is the extracted net photocurrent (Figure 3e), the light intensity, the elementary charge, the dark current, and the effective illuminated area, respectively. In principle, *A* can be estimated by *A* = *nLd* in terms of the number of connected nanowires (*n*), electrode gap (*L*), and average diameter of the nanowires (*d*). However, it is almost impossible to count the number of the nanowires connected by the interdigital electrodes in Figure 3a. Therefore, the effective illuminated area is estimated by summarizing all the area between the electrode gaps, which is ≈4 mm^2^ (Figure S10, Supporting Information). The calculated responsivities and detectivities confirm the sensitivity of these photodetectors to light wavelengths in the 380–980 nm range, covering from the UV, visible, to NIR bands (Figure 3e). At 780 nm, these photodetectors exhibit a maximum responsivity of 1.58 × 10^−5^ A W^−1^ and a detectivity of 1.44 × 109 Jones. Conversely, at 980 nm, the minimum responsivity and detectivity values are 2.98 × 10^−7^ A W^−1^ and 3.25 × 107 Jones, respectively. Note that the effective illuminated area was overestimated during the calculation, the actual responsivity and detectivity could be higher than the values in Figure 3e. The detector has a fast response of <70 ms at different wavelengths of 25.5 mW cm^−2^ (Figure 3f), indicating that the device has a fast response characteristic for the whole wide band of UV–vis–NIR. These findings confirm the preservation of F_16_CuPc's intrinsic properties through the in situ integration strategy employed, underscoring the potential of these devices for detecting broad‐band light across various wavelengths in scientific applications.

**Figure 4:**
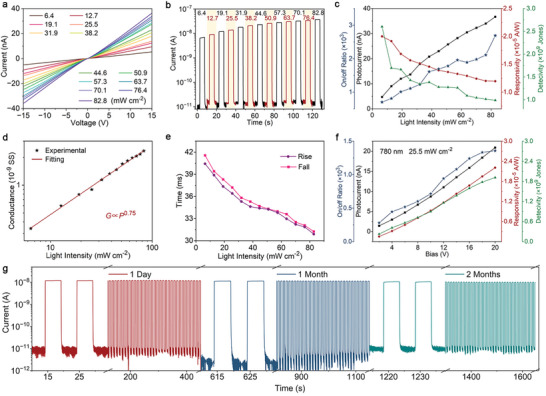
Light intensity‐dependent photoresponse under 780‐nm illumination. a) Voltage‐current curves. b) Photocurrent temporal transients at 15 V bias, the light is modulated with 0.1 Hz on/off frequency. c) Calculated net photocurrents, on/off current ratios, responsivities, and detectivities from (b). d) Photoconductance. e) Rise and fall times. f) Bias‐dependent photocurrent, on/off current ratio, responsivity, and detectivity. g) Long‐term stability of the photoresponse to the on/off‐modulated light (780 nm, 0.1 Hz, 25.5 mW cm^−2^) at 10 V.

In addition to exhibiting the wavelength sensitive photoresponse, the detector also shows high sensitivity to variations in light intensity. As shown in **Figure** **4**a, the current also changes linearly with the applied voltage for each intensity of 780 nm light, suggesting the good ohmic contact between the nanowire and the electrodes, maintained even at the highest intensity examined in this work (82.8 mW cm^−2^). The slope of these curves increases significantly with rising light intensity, suggesting a strong light intensity‐sensitive response. To further investigate the other key parameters of photodetectors, the incident light intensity is modulated using on/off digital signals from a function generator. Typical response currents at a constant bias of 15 V for the illumination of 780 nm light with varying on‐state intensities are plotted in Figure 4b. Again, stable but low dark currents of ≈10^−11^ A were recorded and the photocurrents increase with light intensity. The extracted net photocurrent and on/off ratio both increase with the incident light intensity (Figure 4c). Conversely, the calculated responsivity (specific detectivity) decreases from 2 × 10^−5^ A W^−1^ to 1.2 × 10^−5^ (2.6 × 109 to 9.9 × 108) as light intensity increases (Figure 4c). The plot of photoconductivity versus light intensity follows a power law, *G∝P^γ^* (Figure 4d). The fitting yields a small *γ* value (0.75), indicating a nonlinear increase in photogenerated carriers with absorbed photon flux.^[^ ^39^, ^51^ ^]^ This means the photoconductance is highly related to electron–hole generation, trapping, and recombination within the semiconductor.^[^ ^15^, ^39^ ^]^ The rise and fall times of the photodetector decrease with increasing light intensity. For example, both the rise and decay time reduced from ≈40 to ≈30 ms as the intensity of 780 nm light increases from 6.4 to 82.8 mW cm^−2^ (Figure 4e). The photoresponse of these photodetectors also depends on the applied bias voltage. For instance, under the illumination of 25.5 mW cm^−2^ with 780 nm light, the responsivity and detectivity both increase with bias voltage (Figure 4f). This can be attributed to the combined effect of the increased carrier mobility and density, and the reduced number of trapped carriers at higher bias voltages. The stability and reusability of photodetectors are crucial for practical applications. As shown in Figure 4g, both the dark current and the photocurrent remain stable under long‐term irradiation with on/off‐modulated light. In addition, the photocurrent drops by ≈5% and 12% after one month and two months of storage under ambient conditions, respectively. Figure 4g shows that the dark currents fluctuate between 10^−11^ A and 10^−12^ A. We believe that the main reasons for this small fluctuation are the electrical noise of the test system (e.g., thermal noise, flicker noise) and the changed environmental conditions (e.g., humidity, pressure, and electromagnetic interference).^[^ ^48^ ^]^ However, more detailed work is needed to clarify this issue. Last, these devices still exhibit a similarly fast response of tens of milliseconds after 2 months of storage (Figure S11, Supporting Information). In principle, the stability of the photoresponse can be further improved by appropriate encapsulation, such as the deposition of a protective oxide layer.

**Figure 5:**
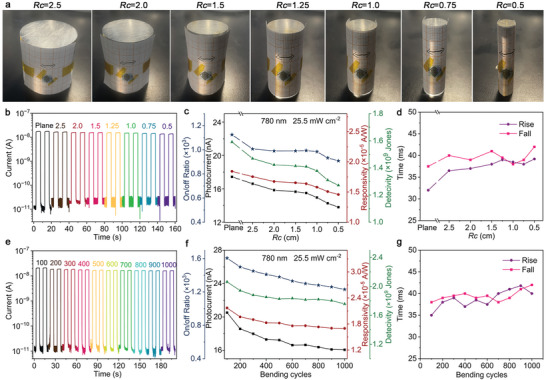
Performance of the monolithic integrated flexible photodetector under different bending conditions. a) Digital photographs of the photodetector when attached to transparent acrylic rods with different radii of curvature (*Rc*). b) Photocurrent temporal transients, c) net photocurrent, calculated on/off current ratio, responsitivity, and detectivity, d) rise time and fall time in response to the same 0.1 Hz on/off modulated 780 nm light (25.5 mW cm^−2^) at 15 V bias voltage under different *Rc*. e) Photocurrent temporal transients, f) net photocurrent, calculated on/off current ratio, responsitivity, and detectivity, g) rise time and fall time in response to the same 0.1 Hz on/off modulated 780 nm light (25.5 mW cm^−2^) at 15 V bias under repeated bending cycles.

To assess the performance stability of the photodetector under varying bending conditions, the device was initially mounted to transparent acrylic rods with different radii of curvature (*Rc*) (**Figure** **5**a). As depicted in Figure 5b, the dark currents remain at a level as low as 10^−11^ A, while the on‐state photocurrent decreased from 17.4 to 13.8 nA as *Rc* decreased continuously to 0.5 cm. Despite this reduction, these photocurrent temporal transients maintain a consistent square shape, indicating a well preserved photoresponse capability. The minimum *Rc* of 0.5 cm suggests that the electrode contact remained stable and reliable even under significant bending stress. Analysis of these curves revealed a decrease of 20.8% in net photocurrent, 19.1% in on/off current ratio, 21.9% in responsivity, and 19.9% in detectivity of the detector (Figure 5c). These declines can be attributed to two primary factors. First, the increased bending may have damaged some nanowires, altering their contact with the electrodes. Additionally, the effective light‐absorption region changes significantly under high bending conditions. Figure 5d illustrates that both the rise and fall times exhibited negligible variations with increasing bending, suggesting that the reduced absorption area due to bending was the primary factor leading to the decrease in on‐state photocurrent and subsequent reductions in other performance parameters. In addition, the detector maintains its performance even after a large number of bending cycles. Figure 5e shows that, despite a decrease in on‐state photocurrent from 20.5 to 16.1 nA after 1000 bending cycles, the dark current and square shape of the curves remained consistent, indicating a consistent photoresponse. After 1000 bending cycles, the photocurrent, on/off ratio, responsivity, and detectivity decreased by 21.6%, 23.3%, 20.8%, and 14.3%, respectively (Figure 5f), while the rise time and fall time exhibited negligible change (Figure 5g).

**Figure 6:**
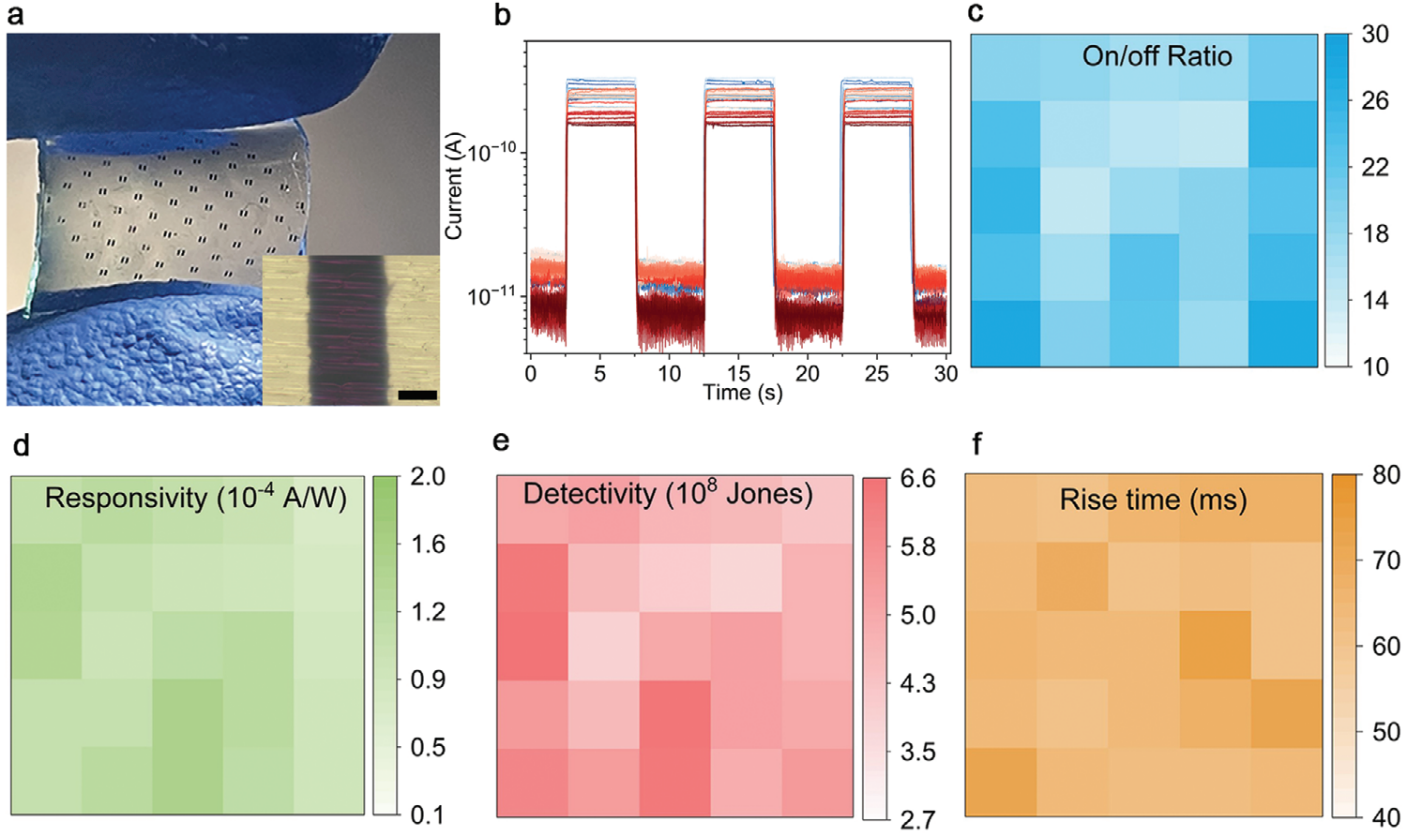
On‐chip flexible photodetector arrays from the guided F_16_CuPc nanobelts on PDMS films and their performance. a) Digital photography of all 9 × 9 photodetector cells, the inset shows the optical microscopy image of the nanowires bridged between two electrodes and the scale bar is 20 µm. b–f) Photoresponse at 15 V bias voltage and illumination of 780 nm, 0.1 Hz, 25.5 mW cm^−2^, b) photocurrent temporal transients, distribution of on/off current ratio (c), responsivity (d), detectivity (e), rise time (f).

In spite of the successful development of the aforementioned flexible monolithic photodetector with high bending stability, the integration of self‐aligned nanowires as on‐chip arrays of photodetectors is also achievable. A 9 × 9 array of photodetectors was fabricated by employing a shadow mask with designed micro‐holes to shield the nanowires during the deposition of gold electrodes (**Figure** **6**a). In this study, the electrodes’ dimensions are 200 µm × 100 µm, with a 40 µm gap between adjacent electrodes (inset in Figure 6a). By adjusting the density of holes in the mask, the number of photodetector cells can be scaled up as needed, offering an additional advantage compared to using nanowires transferred on flexible films. As shown in Figure 6b, all the photodetector cells show consistent photocurrent transients upon exposure to 780 nm light. Their net photocurrent varies between 0.16 and 0.34 nA at a bias voltage of 15 V, averaging at 0.24 nA (Figure S12, Supporting Information). Considering their dark currents, the on/off current ratio falls within the 14.2–28.1 range, with an average of 20.1 (Figure 6c; Figure S13, Supporting Information). Compared to the monolithic photodetector with finger electrodes, the average photocurrent and the resulting on/off current ratio of the arrayed photodetectors are almost two orders of magnitude lower. This is expected due to the larger number of nanowires connected in parallel in the former detector. It is well known that the total current in a circuit can multiply with an increase in parallel resistance. These findings underscore another advantage of guided nanowires in enhancing the on/off current ratio of a photodetector, particularly important for high resistance nanowires, such as most organic nanowires.

To simplify the calculation of their responsivity and detectivity, the gap area between two adjacent electrodes (200 µm × 40 µm) was used as the effective illumination area (*A*). Therefore, the calculated responsivity (Figure 6d) and detectivity (Figure 6e) are 0.73 × 10^−4^–1.55 × 10^−4^ A W^−1^ and 3.75 × 108–6.55 × 108 Jones, respectively, with mean values of 1.11 × 10^−4^ A W^−1^ and 5.12 × 108 Jones, respectively. The significant variation in photocurrent, on/off current ratio and detectivity primarily stems from differences in the number and radius of the bridged nanowires and variations in electrical contact conditions. Further investigation is needed to address these challenges. In contrast, the differences in response speed are relatively narrow. For instance, detector cells exceeding 80% exhibit rise and fall times of 60–70 ms (Figure 6f; Figure S14, Supporting Information). This observation is reasonable as the response speed is primarily influenced by the quality of the nanowires and the lighting conditions rather than the number and radius of the bridged nanowires. This advantage is particularly important for imaging applications that require a larger number or higher density of photodetector arrays. Last, compared to the reported photodetectors fabricated using different F_16_CuPc structures and flexible photodetectors made of aligned CuPc nanowires, these in situ integrated flexible nanowire photodetectors exhibit similar photoresponse capabilities across the broad UV–Vis–NIR spectrum (**Table** **1**). This demonstrates the feasibility of our in situ integration strategy. In addition to the advantage of in situ integration of nanowires on flexible films, these devices stand out for their fast response speed and stable performance even when subject to severe bending.

**Table 1:** Performance comparison between our flexible photodetectors and the reported F_16_CuPc photodetectors and two representative flexible photodetectors made of organic materials.

Materials	Flexibility	Methods	Wavelength [nm]	Responsivity [A W^−1^]	Detectivity (Jones)	Speed [ms]	Ref.
F_16_CuPc film	No	Vacuum deposition	–	2 × 10^−3^	–	100–200	[52]
Pentacene/F_16_CuPc	No	Thermal evaporation	300–900	3 × 10^−3^	10^10^	410–420	[53]
Single F_16_CuPc nanowires	No	Post‐growth transfer	589–940	5.5 × 10^−5^	–	448	[54]
Single F_16_CuPc nanoflakes	No	Exfoliation	300–750	19	8 × 10^12^	36–72	[55]
F_16_CuPc nanoribbon arrays	No	In situ integration	405–850	1 × 10^−3^	3 × 10^9^	30–80	[37]
CuPc nanowire arrays	Yes	In situ integration	405–980	5.5 × 10^−4^	8 × 10^8^	100–5000	[39]
CuPc nanowire arrays	Yes	Post‐growth transfer	500–800	–	–	>100 s	[15]
P3HT/CCS hybrid films	Yes	Post‐growth transfer	365	240	9 × 10^12^	12–150	[56]
CH_3_NH_3_PbI_3_/C8‐BTBT	Yes	Spin‐coating	532	8.1	2.17 × 10^12^	7.1	[57]
F_16_CuPc nanowire arrays	Yes	In situ integration	380–980	1.1 × 10^−4^	2.6 × 10^9^	25–67	This work^a)^

^a)^ The listed responsivity and detectivity are the maximum values we have currently obtained.

## 3 Conclusion

In summary, the innovative approach presented in this study successfully addressed the longstanding challenge of direct growing highly aligned nanowires on amorphous flexible surfaces. By replicating CD nanogrooves on a PDMS surface, large‐area self‐aligned nanowires made of F_16_CuPc and various metal phthalocyanine molecules were seamlessly incorporated into monolithic flexible photodetectors. These devices exhibited remarkable characteristics, including rapid response times and long‐term stability under UV–Vis–NIR light. In particular, they demonstrated exceptional resistance to bending stress, maintaining similar photoresponse even after 1000 bending cycles with a curvature radius as small as 0.5 cm. Additionally, the consistent sensitivity and response times observed across a photodetector array integrated with self‐aligned nanowires highlight the potential for on‐chip integration of flexible photodetectors based on guided nanowires. This study not only validates the feasibility of guided nanowire growth on polymer surfaces by replicating CD nanogrooves but also emphasizes the promising prospects for on‐chip integration of flexible nanowire‐based photodetectors, representing a significant advancement in the realm of flexible electronics.

## 4 Experimental Section

### Replication of CD Nanogrooves onto a Flexible Film

The dye layer and the reflective layer of as‐purchased commercial CD (Maxell) were first separated mechanically from the underlying polycarbonate substrate. The exposed polycarbonate substrate was then placed in a petri dish with the grooves facing upward. The Sylgard 184 silicone elastomer base (Dow Corning) was then mixed with the Sylgard 184 silicone elastomer curing agent (Dow Corning) at a 10:1 ratio, stirred thoroughly, and degassed in vacuum at room temperature until all air bubbles were eliminated. Next, the PDMS mixture was casted onto the exfoliated polycarbonate surface to a desired thickness. After curing at 60 °C for 2 h on a vacuum drying oven, the PDMS was peeled off from the polycarbonate plate. A mixture of 10 g of polyvinyl alcohol particles (polyvinyl alcohol 4–88, purchase from Aldrich) and 50 mL of deionized water was stirred magnetically at 90 °C until the particles were completely dissolved to obtain a PVA film precursor. The PVA precursor was casted onto the exfoliated polycarbonate surface to a desired thickness. After curing in air at room temperature for >8 h, the PVA film was peeled off from the polycarbonate plate.

### Guided Growth of Nanowires on a Flexible PDMS Film

The growth was conducted in a horizontal tube furnace system with dual temperature zones (Shanghai Weixing Furnace, TF1200‐60). A quartz boat filled with 10 mg of precursor powder (95% F_16_CuPc, 97% CoPc from Sigma–Aldrich, 95% CuPc, 96% FePc, 95% NiPc, 97% ZnPc from Alfa Aesar) and the PDMS films with replicated nanogrooves were sealed in a quartz tube by flanges. The sealed tube was then mounted on the tube furnace, with the boat locating at the center of a heating zone, and the PDMS films placing 18 cm downstream away from quartz boat. Before heating up, the air inside the tube furnace was pumped out (below 10 mbar), followed by the injection of nitrogen to reach atmospheric pressure, and then pumped below 10 mbar again. The sealed tube was purged three times to ensure an inert gas environment for nanowires growth. The source temperature was ramped up to 460 °C in 30 min, and the substrate temperature was ramped up to 260 °C in 30 min. During nanowire growth, the gas flow rate was set to 250 sccm, and the pressure maintained at 25 mbar. After 120 min of growth under these conditions, the PDMS film was taken out from the heating zone immediately and cooled down to room temperature rapidly.

### Structural Characterizations

SEM observations were performed with a Zeiss Gemini 500 (operated at 2 kV). AFM measurements were performed with Bruker Multimode 8. XRD measurements were carried out on a BRUKER D8 ADVANCE with a Cu Kα (λ  =  1.5406 Å). The absorption spectra of F_16_CuPc nanowires were measured by UV–vis spectrometer (SHIMADZU UV‐2600). Raman shift is recorded on a confocal Raman system (Renishaw inVia with a 785 nm laser as excitation.)

### Fabrication and Measurement of Flexible Photodetectors

The gold interdigital electrodes and arrays of microscale electrodes were directly deposited onto the PDMS surface grown with horizontally‐oriented F_16_CuPc nanoribbons using a metal mask by thermal evaporation coating instrument (INFICON SQC‐310, Shenyang Kecheng Vacuum Tech Co, Ltd). All measurements were done under the ambient environment at room temperature using a CPPS03‐DN probe station (Jinan Chuangpu Co. Ltd) with a Keithley 2612B System Source Meter. Lasers (380, 405, 450, 520, 635, 780, 808, 850, 980 nm) were used to illuminate the device. For on/off current measurements, the laser was periodically switched on and off by a dual‐channel arbitrary function generator (Tektronix AFG1062). Finally, the bending tests were carried out under various bending radii of curvature (*Rc* = 2.5, 2.0, 1.5, 1.25, 1.0, 0.75, 0.5 cm).

## Conflict of Interest

The authors declare no conflict of interest.

## Supporting information

Supporting Information

## Data Availability

The data that support the findings of this study are available from the corresponding author upon reasonable request.
