# Supplementary material for: Replicating CD Nanogrooves onto PDMS to Guide Nanowire Growth for Monolithic Flexible Photodetectors with High Bending‐Stable UV–vis–NIR Photoresponse
Source: Adv Sci (Weinh). 2024 Jun 20;11(32):2403870. doi: 10.1002/advs.202403870 (PMC11348143; doi:10.1002/advs.202403870)
Supplement: Supplementary file 1 — Supporting Information [file ADVS-11-2403870-s001.docx]

Supporting Information

**Replicating CD Nanogrooves onto PDMS to Guide Nanowire Growth for Monolithic Flexible Photodetectors with High Bending-Stable UV-Vis-NIR Photoresponse**

*Hanyu Liu, Wei Zhou*, Xiangtao Chen, Pingyang Huang, Xingyu Wang, Guofu Zhou, Jinyou Xu**

Guangdong Provincial Key Laboratory of Optical Information Materials and Technology & Institute of Electronic Paper Displays

South China Academy of Advanced Optoelectronics

South China Normal University

Guangzhou 510006, People’s Republic of China

Email: zwei@m.scnu.edu.cn; jinyou.xu@m.scnu.edu.cn


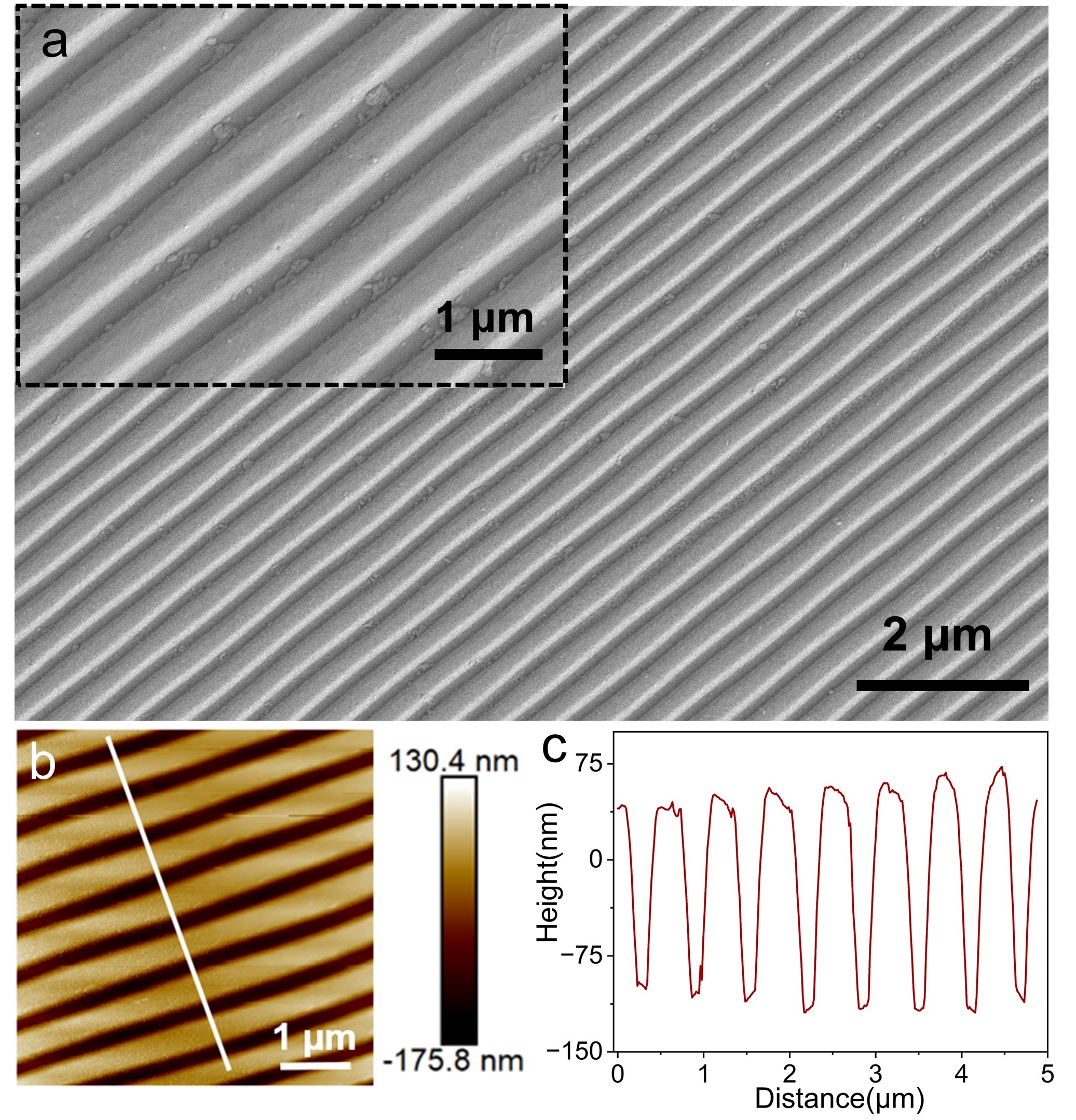


**Figure S1**. SEM (a), AFM (b) and heigh profile (c) of the exfoliated CD polycarbonate layer with aligned periodic nanogrooves.


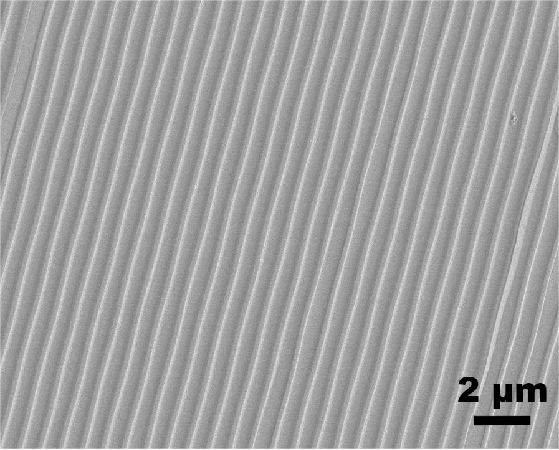


**Figure S2**. SEM image of the PVA film replicated with CD nanogrooves.


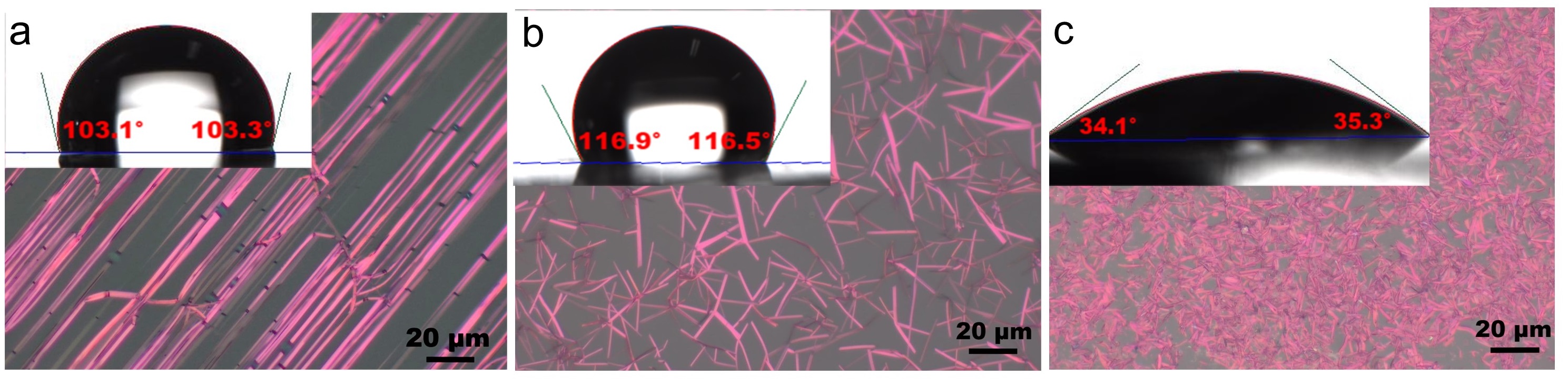


**Figure S3**. Optical microscope images of the nanowires grown on the hydrophobic PDMS film with replicated CD nanogrooves (a), on the hydrophobic flat PDMS film (b), and on the hydrophilic PDMS film (c). The insets are the water contacting angle on these films.


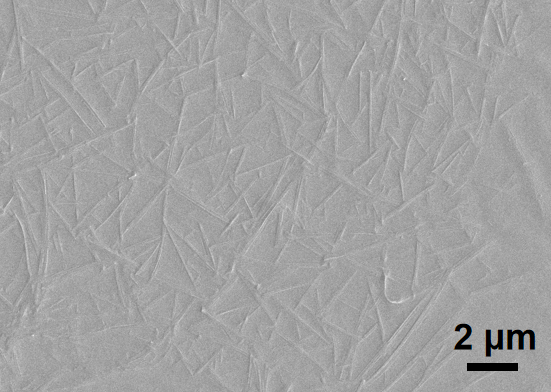

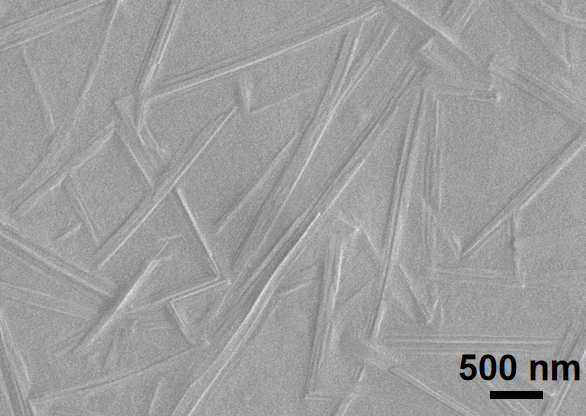


**Figure S4**. SEM images of the F_16_CuPc nanobelts grown on a flat PDMS film without replicating the CD nanogrooves.


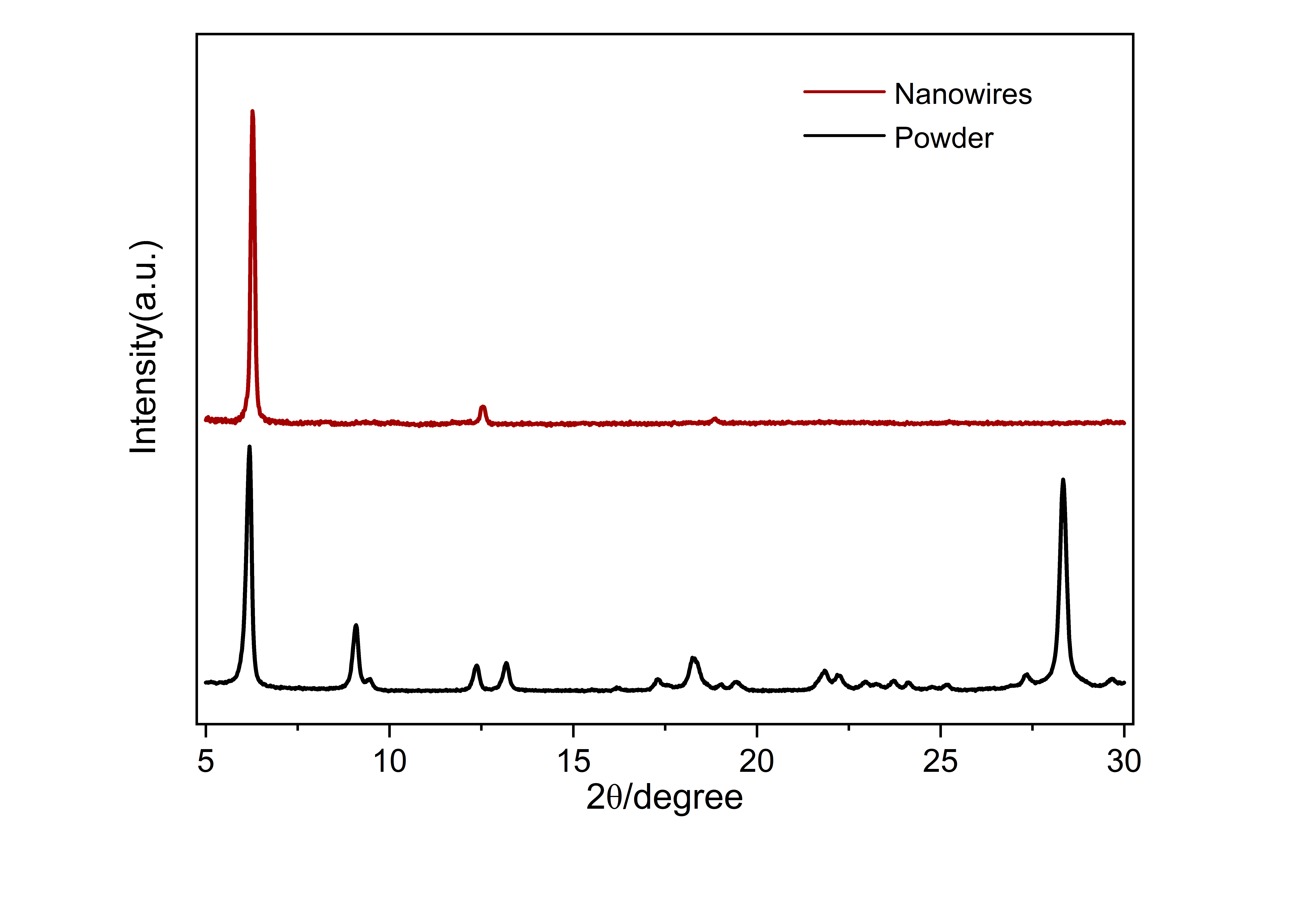


**Figure S5**. XRD of as-grown nanobelts and the powder used for evaporation.


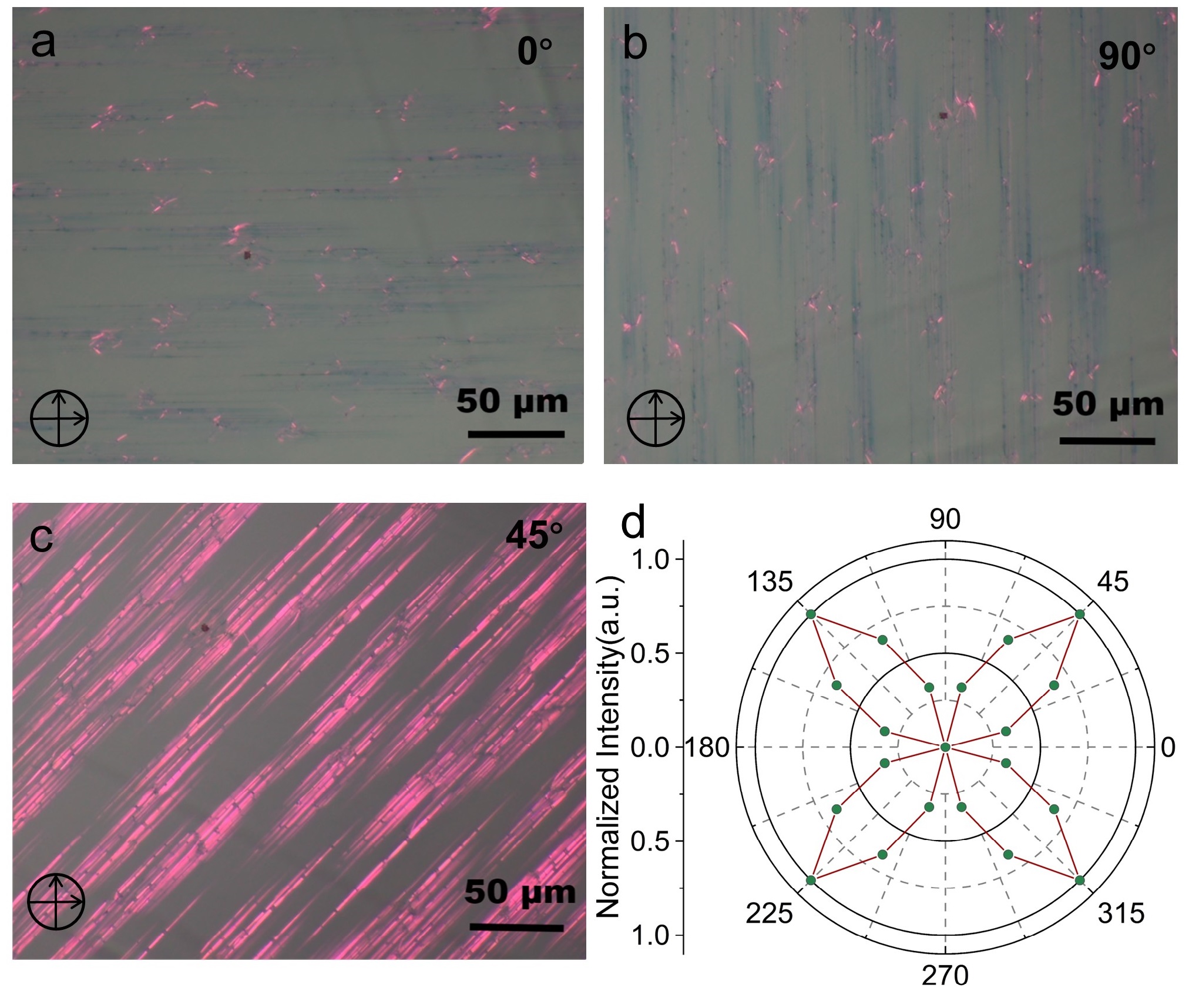


**Figure S6**. Cross-polarized optical microscopy images under different polarized angels (a-c) and (d) normalized intensity of the aligned F_16_CuPc nanowires.


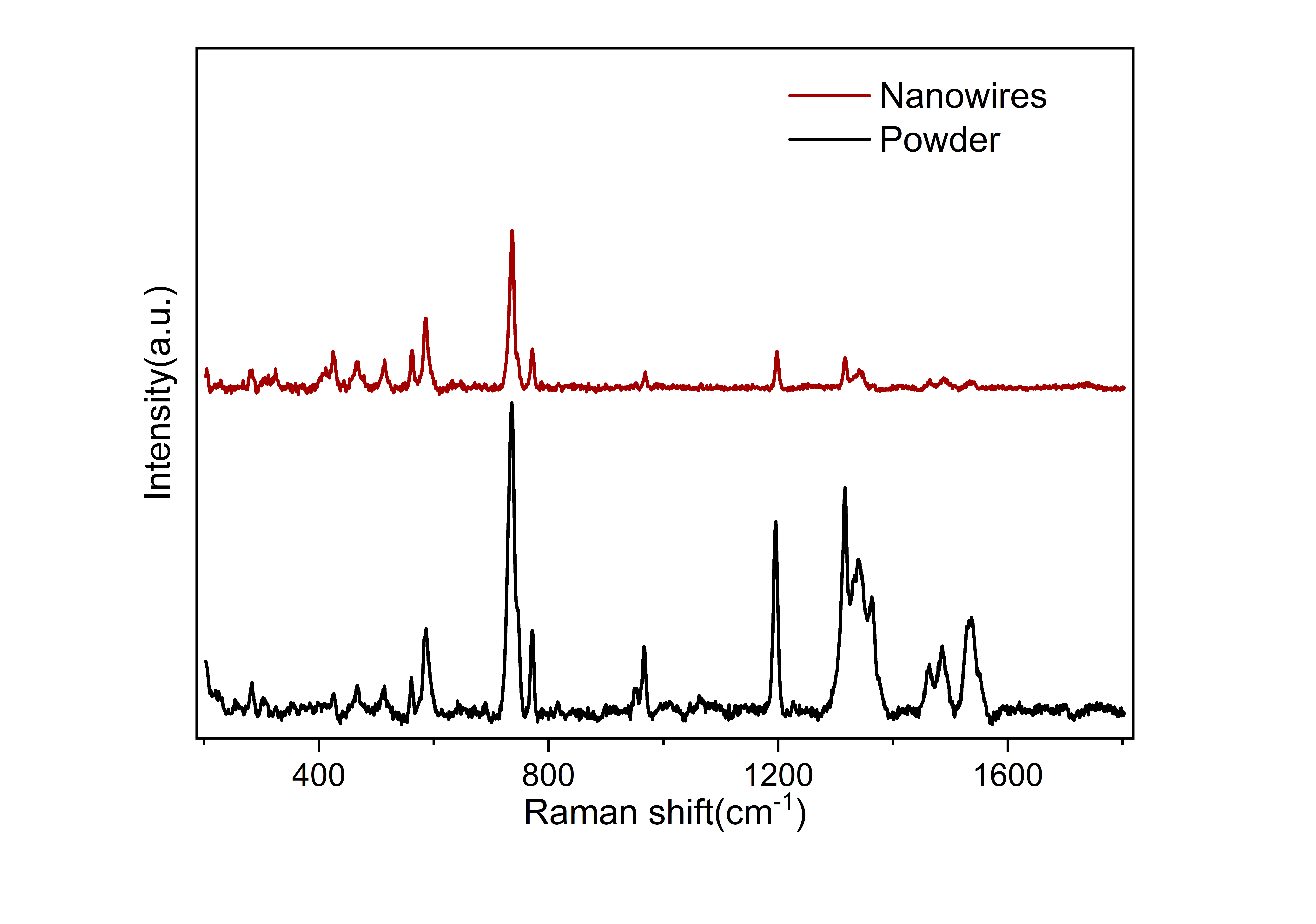


**Figure S7**. Raman spectra of as-grown nanobelts and the powder used for evaporation.


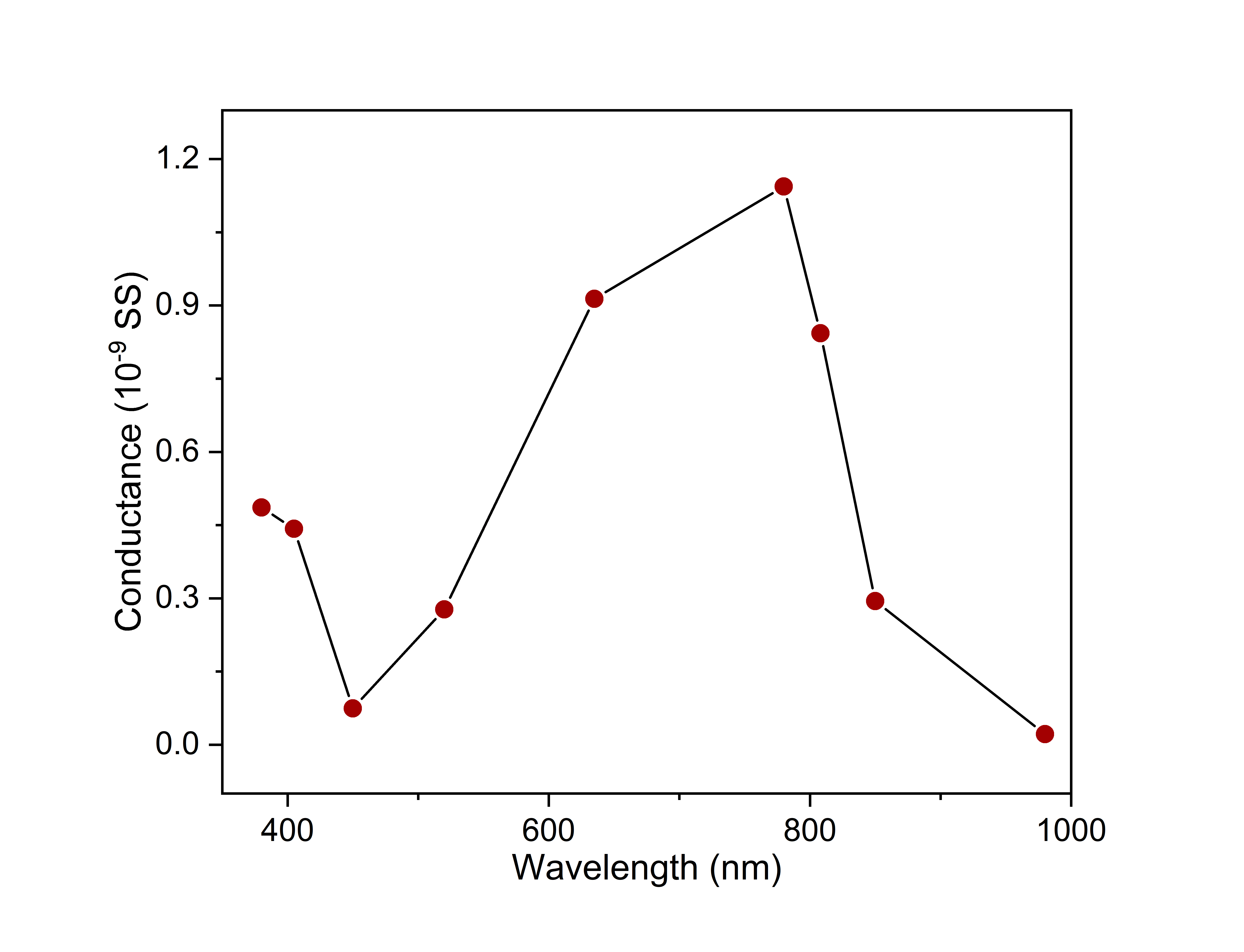


**Figure S8**. Conductivity versus wavelength, calculated from the current-voltage curves in Figure 3c.


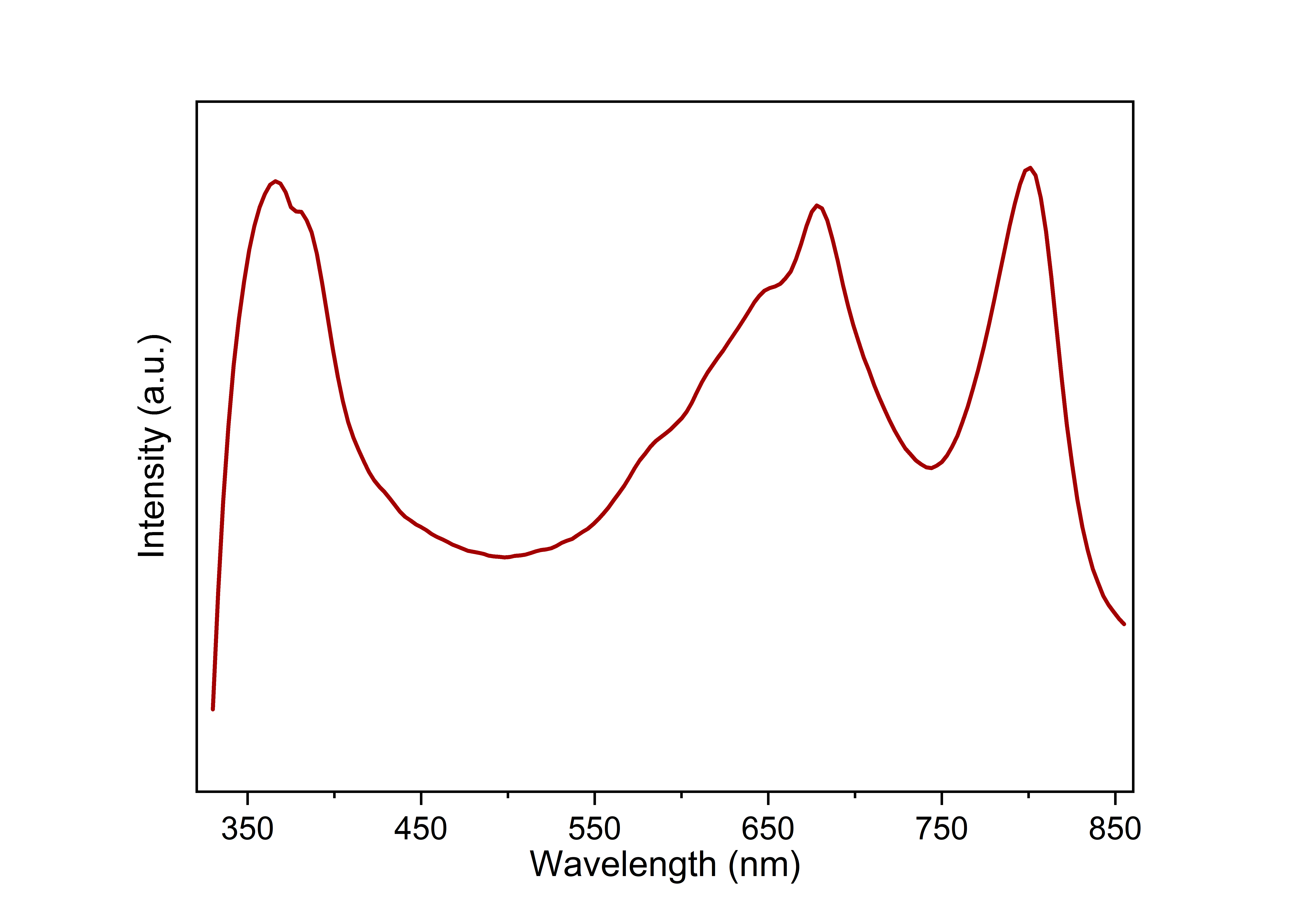


**Figure S9**. Absorption spectrum of the guided F_16_CuPc nanobelts.


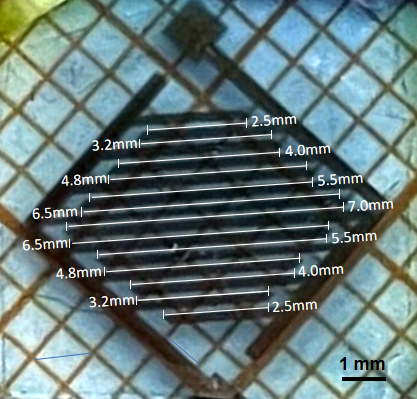


**Figure S10**. Estimation of effective illumination area.


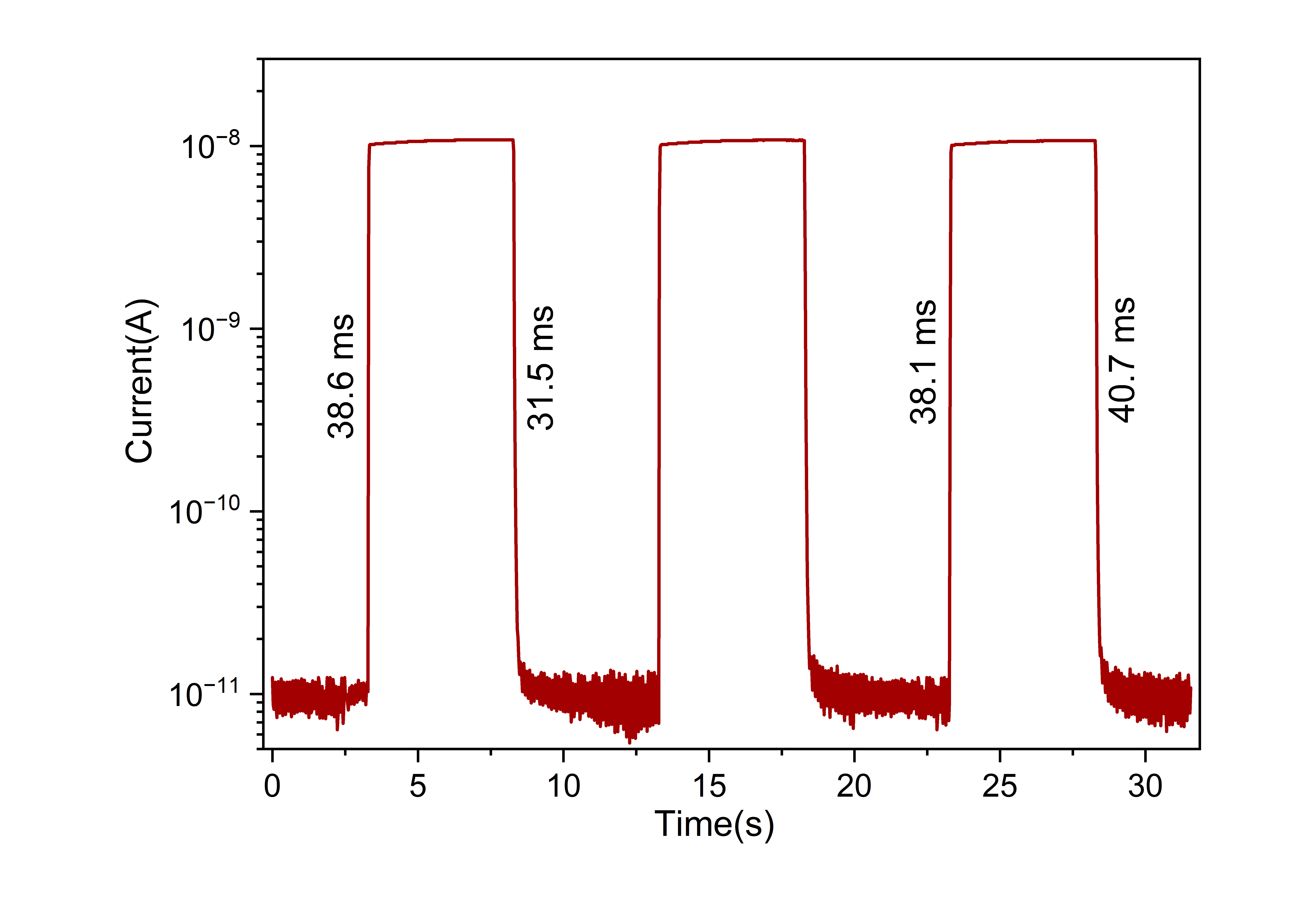


**Figure S11**. Response times of the photodetector after 2 months of storage.


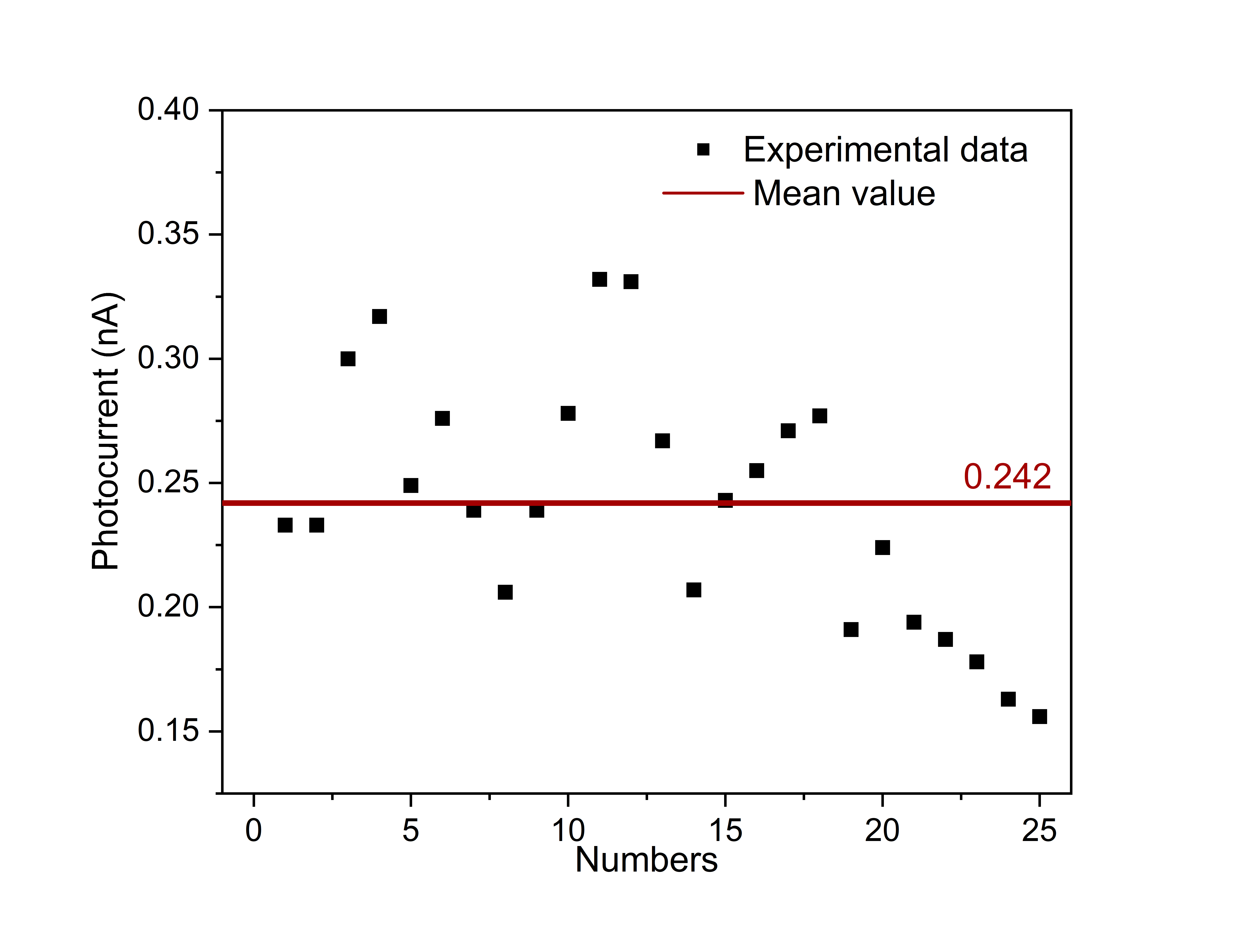


**Figure S12**. Net photocurrent of 25 photodetector cells.


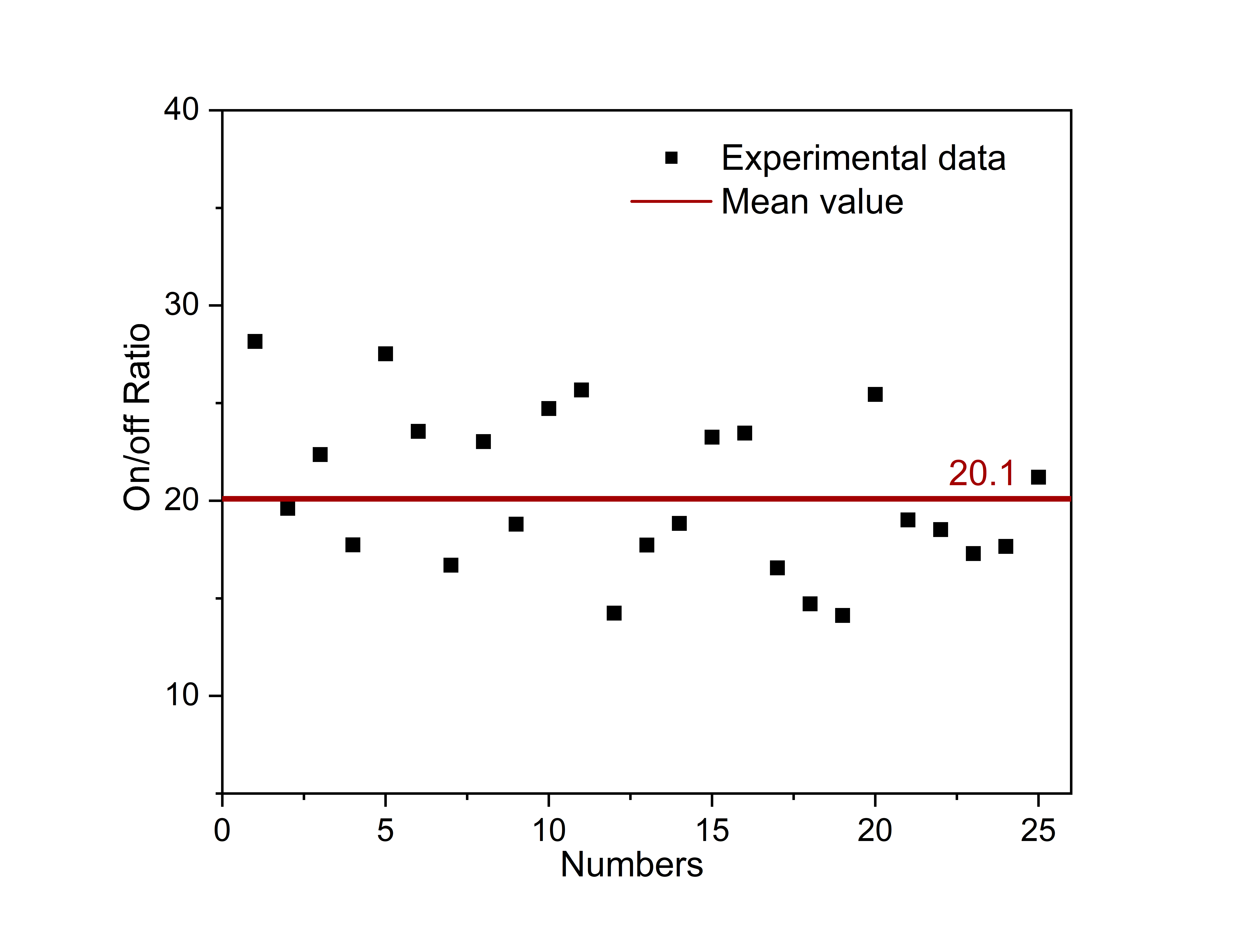


**Figure S13**. On/off current ratio of 25 photodetector cells.


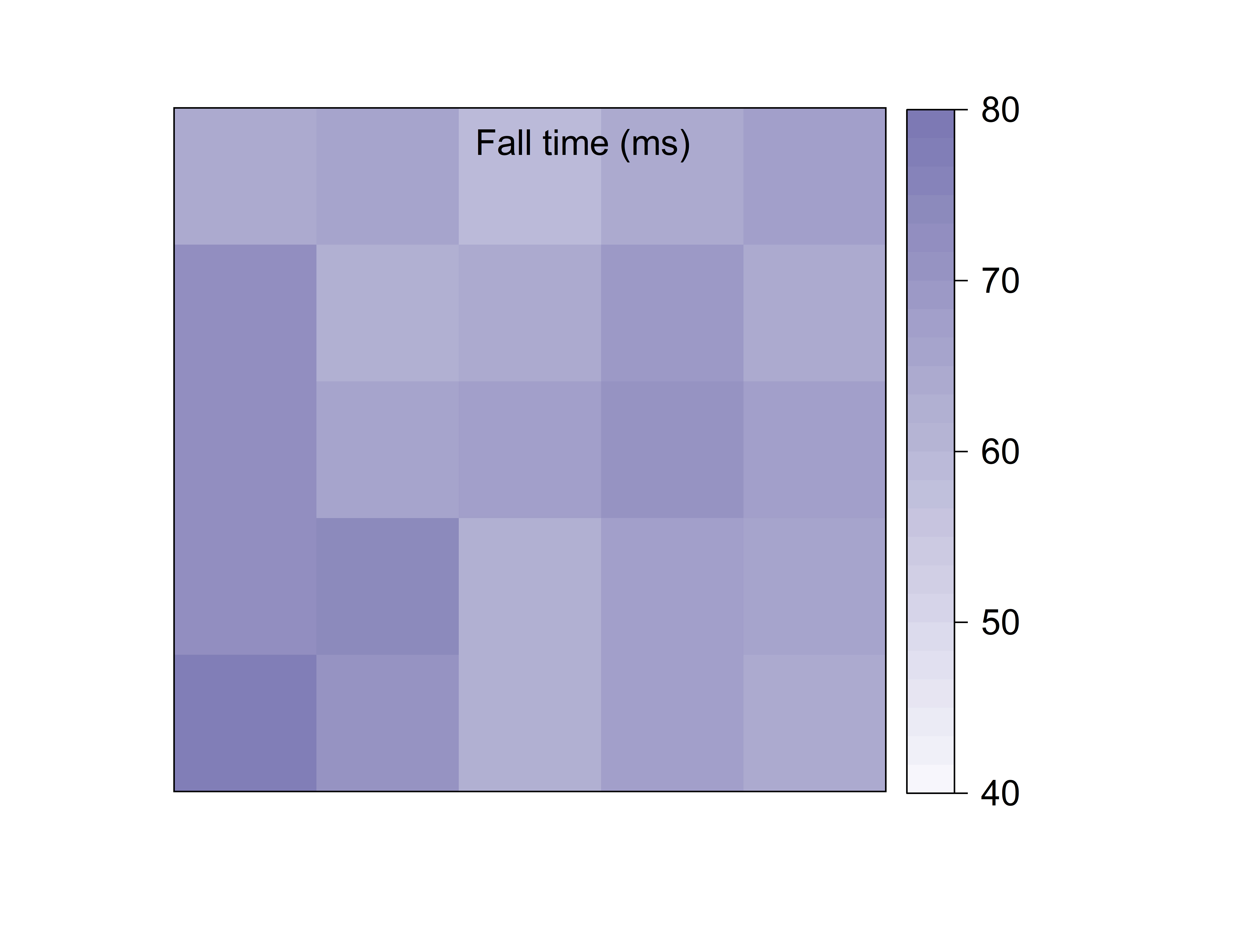


**Figure S14**. Distribution of fall time at 15 V bias, under illumination of 780 nm (25.5 mW cm^-2^).
